# Can Transcranial Electrical Stimulation Localize Brain Function?

**DOI:** 10.3389/fpsyg.2019.00213

**Published:** 2019-02-19

**Authors:** Anke Ninija Karabanov, Guilherme Bicalho Saturnino, Axel Thielscher, Hartwig Roman Siebner

**Affiliations:** ^1^ Danish Research Centre for Magnetic Resonance, Centre for Functional and Diagnostic Imaging and Research, Copenhagen University Hospital Hvidovre, Hvidovre, Denmark; ^2^ Department of Electrical Engineering, Technical University of Denmark, Copenhagen, Denmark; ^3^ Department of Neurology, Copenhagen University Hospital Bispebjerg, Copenhagen, Denmark; ^4^ Institute for Clinical Medicine, Faculty of Health Sciences and Medicine, University of Copenhagen, Copenhagen, Denmark

**Keywords:** TES, cognition, electric field modeling, non-specific effects, transcranial alternate current stimulation, transcranial direct current stimulation, dosing

## Abstract

Transcranial electrical stimulation (TES) uses constant (TDCS) or alternating currents (TACS) to modulate brain activity. Most TES studies apply low-intensity currents through scalp electrodes (≤2 mA) using bipolar electrode arrangements, producing weak electrical fields in the brain (<1 V/m). Low-intensity TES has been employed in humans to induce changes in task performance during or after stimulation. In analogy to focal transcranial magnetic stimulation, TES-induced behavioral effects have often been taken as evidence for a causal involvement of the brain region underlying one of the two stimulation electrodes, often referred to as the active electrode. Here, we critically review the utility of bipolar low-intensity TES to localize human brain function. We summarize physiological substrates that constitute peripheral targets for TES and may mediate subliminal or overtly perceived peripheral stimulation during TES. We argue that peripheral co-stimulation may contribute to the behavioral effects of TES and should be controlled for by “sham” TES. We discuss biophysical properties of TES, which need to be considered, if one wishes to make realistic assumptions about which brain regions were preferentially targeted by TES. Using results from electric field calculations, we evaluate the validity of different strategies that have been used for selective spatial targeting. Finally, we comment on the challenge of adjusting the dose of TES considering dose–response relationships between the weak tissue currents and the physiological effects in targeted cortical areas. These considerations call for caution when attributing behavioral effects during or after low-intensity TES studies to a specific brain region and may facilitate the selection of best practices for future TES studies.

## Introduction

Transcranial electric stimulation (TES) applies weak electric currents to non-invasively stimulate the human brain. TES uses either constant, oscillating, or randomly alternating currents to interact with membrane potentials. The application of constant currents is called transcranial direct current stimulation (TDCS) (Nitsche and Paulus, 2000; Stagg et al., 2018), the application of sinusoidal currents is referred to as transcranial alternating current stimulation (TACS) (Antal et al., 2008; Herrmann et al., 2013), and stimulation at randomly alternating amplitudes and frequencies is labeled transcranial random noise stimulation (TRNS) (Terney et al., 2008). TES enjoys high popularity in human neuroscience because of its low cost and high availability. TES is easy to apply through quickly mountable scalp electrodes. Most TES studies apply low-intensity currents (≤2 mA) *via* a pair of large pad electrodes (i.e., bipolar montage). In recent years, multi-electrode arrangements have been introduced in TES experiments to achieve more focal stimulation and supplement the prevailing use of bipolar montages (Douglas et al., 2015; Gallo et al., 2018). The use of TES in cognitive neuroscience continues to be very popular with more than 50 new studies listed in PUBMED in the first half of 2018. All these studies explored the effects of TES on task performance in healthy volunteers, and the majority of studies reported TES-related alterations in performance in the task of interest.

The neurobiological effects of TES depend on the magnitude of the electrical field, its direction with respect to the stimulated neural target structure, and the conductivity of the stimulated tissue. In contrast to transcranial magnetic stimulation (TMS), the electric fields induced by TES are too weak to sufficiently depolarize cortical neurons to trigger action potentials: Invasive recordings and electric field modeling have provided converging evidence that electrical fields induced by TES do not exceed 1 V/m in the brain (Datta et al., 2009; Opitz et al., 2015, 2016; Huang et al., 2017). At these electrical field strengths, the injected current may induce minor shifts in the membrane potential and result in modification of intrinsic neuronal network activity, for instance by triggering stochastic resonance, rhythm resonance, or a bias in the timing of intrinsic spiking.

The mechanisms by which TDCS, TACS, and TRNS interact with intrinsic brain activity are still incompletely understood (Ali et al., 2013; Hanslmayr et al., 2014; Widge, 2018), but TES methods have been widely used in healthy individuals to change participants’ performance in well-defined experimental tasks (Stagg and Nitsche, 2011; Berryhill and Martin, 2018; Polania et al., 2018). Changes in task performance are not only observed during the administration of TES (online) but are also found after the end of a TES session (offline), suggesting that TES may have lasting effects on specific brain functions.

The effects of TES on behavior have prompted scientists to make inferences about the functional localization of brain functions. In analogy to focal TMS, TES-induced behavioral effects have often been taken as evidence for a causal involvement of the brain region underlying one of the TES electrodes. This line of reasoning has been supported by a seminal paper by Nitsche and Paulus (2000), which tested excitability changes of the primary motor cortex (M1) caused by a bipolar TDCS montage with one electrode covering the primary motor hand area (M1-HAND) and the other covering the contralateral supraorbital area. Using motor evoked potentials (MEPs) as a proxy for M1 excitability, the study demonstrated that anodal currents injected at the M1 electrode increased the MEP size, while cathodal currents decreased the MEPs. As such modulations of MEP amplitude did not occur when the “active” electrode was placed anterior or posterior to M1-HAND, the authors concluded that the excitability modulating effects of TES were confined to the area under the electrode. It is important to note here that MEP-based studies cannot be used to test for cortical TES effects outside of the motor cortex, as the measure is only sensitive to excitability changes within the corticospinal tract. Since then, these results have been used nevertheless by many TES studies in the field of cognitive neurosciences as an argument for the focality of TES effects when placing one electrode (sometimes called the active electrode) over the cortical area of interest and the other electrode (sometimes called the reference or return electrode) over a remote site on the scalp. They have used the underlying assumption that behavioral effects induced by bipolar TES could reveal the functional role of the cortical region underlying the “active” electrode with little regard to the location and size of the other electrode and the current flow in the tissue between the two electrodes (e.g., Ruff et al., 2013; Weiss et al., 2013; Pope et al., 2015). This line of reasoning is still prevailing in TES studies on human cognition (Choy et al., 2018; Folmli et al., 2018; Peled-Avron et al., 2018).

The notion that the cortex underlying one of the two electrodes corresponds to the main site of TES action on brain networks is too simplistic and incorrect in most instances. Simulation studies that model the electric field distributions in the brain have provided converging evidence that the properties (e.g., size) and location of both electrodes, along with the geometry and conductivity of the tissue compartments determine which brain areas are preferentially targeted (Opitz et al., 2015; Saturnino et al., 2015). The complex relationships between electrode placement and the location as well as spatial spread of TES currents (Lang et al., 2005) are much more acknowledged: The inherent ambiguities of bipolar montages in terms of site-specific targeting render it problematic to attribute behavioral and physiological TES effects to a specific cortical target region (e.g., the cortex underlying one of the electrodes), limit the mechanistic interpretability of TES studies, and contribute to growing concerns about reliability and reproducibility (Horvath et al., 2015). The complexity and non-focality of TES effects are also corroborated by human brain mapping studies, showing that neural effects of bipolar TES are more prominent in remote brain regions rather than selectively interacting with the superficial cortex that is underlying one specific electrode (Lang et al., 2005; Fiori et al., 2018; Fonteneau et al., 2018).

In this review, we discuss the utility of bipolar low-intensity TES to localize human brain function. In the first part, we summarize physiological substrates that constitute peripheral targets for TES and may mediate subliminal or overtly perceived peripheral stimulation. We make the point that peripheral co-stimulation may contribute to the behavioral effects of TES and discuss how well they are controlled for by “sham” stimulation. In the second section, we discuss biophysical properties of TES, which need to be considered if one wishes to make realistic assumptions about which brain regions were actually targeted by TES. Using results from electric field calculations, we evaluate the validity of different strategies that have been used for selective spatial targeting. In the third part, we address the challenge of adjusting the dose and summarize current knowledge about the dose–response relationship of physiological effects in targeted cortical areas.

## Non-Specific Stimulation Effects

The sensory side effects of TES include itching, tingling, and burning sensations under the electrode. Depending on the electrode montages, vertigo and visual phenomena such as phosphenes are also common (Matsumoto and Ugawa, 2017). These non-specific side effects are caused by concomitant stimulation of afferent nerves and sensory organs (Antal et al., 2017; Matsumoto and Ugawa, 2017) and present a challenge for TES studies for two reasons: First, the conscious perception of co-stimulation can change overall alertness to a task and induce placebo effects. Second, even if without conscious perception, co-stimulation of afferent nerve endings allows for the possibility of an indirect brain modulation through sensory stimulation. While the conceptual considerations on sensory co-stimulations are applicable to both TACS and TDCS, the current waveform (constant or oscillating) affects the conscious perception of this stimulation: Shifting currents cause consciously detectible sensory phenomena, and hence, the influence of sensory co-stimulation is better recognized for TACS. This is why we will discuss the two techniques separately in the paragraphs below.

## Transcranial Direct Current Stimulation

Due to the relatively quick shifts in current at the beginning of the experiment, perceptual side effects are primarily reported during the start of TDCS. An attempt of matching these side effects of non-cortical co-stimulation is made by almost every study. Minimizing the electrode-skin impedance (Davis et al., 2013; Antal et al., 2017) and using long current ramp-up and ramp-down periods (Ambrus et al., 2012) seek to minimize the sensory side effects during the active experimental conditions. The remaining sensations are often matched by a sham-control condition in which electrical current is only shortly ramped up and down at the beginning of the stimulation protocol (Ambrus et al., 2012). This type of sham stimulation has a negligible impact on the cortex and matches the sensory perception during the active conditions relatively well, as the conscious sensory effects during TDCS wane shortly after stimulation onset. Sham control has shown to effectively match perception of co-stimulation for intensities up to 1 mA (Brunoni et al., 2011) even though more recent work suggests that blinding may be compromised even at these low intensities (Turi et al., 2018). At higher stimulation intensities (≥1.5 mA), the efficacy of sham control is clearly reduced, as studies have consistently reported stronger subjective sensations in the active conditions (Kessler et al., 2012; O’Connell et al., 2012). The use of more focal pseudo-unipolar montages seems to reduce the concomitant stimulation of afferent nerves and sensory organs, and sham control with more focal electrodes may be effective up to 3 mA (Garnett and den Ouden, 2015; Reckow et al., 2018).

While sham controls may avoid placebo effects and top-down attentional modulation, they do not mitigate the issue of indirect brain stimulation *via* peripheral input. Depending on the montage, cutaneous receptors, cranial nerves as well as other sensory organs such as the retina or the vestibular organ may be stimulated. Even when remaining below the perceptual threshold, the peripheral stimulation may modulate the input the brain receives *via* these routes. There is little research on the effects of concomitant nerve stimulation, but one current study found that anodal TDCS over the primary motor cortex (montage cathode/anode: Fp3/C3) modulated the trigeminal-facial reflex circuit (Cabib et al., 2016). The observed modulation was greater ipsilateral to the electrodes, indicating that the effect was at least partially mediated through direct trigeminal stimulation.

Especially in TDCS montages that place the return electrode over the mastoid (e.g., (Chrysikou et al., 2013; Van’t Wout et al., 2017), the peripheral vestibular system is also likely co-stimulated. In fact, a specific form of direct current application called galvanic vestibular stimulation uses electric currents with similar strength (0.5–2 mA, DC or AC) through electrodes over the mastoid to polarize the otoliths and the semicircular canal of the vestibular nerve either unilaterally or bilaterally (Utz et al., 2010). Vestibular stimulation, even at a sub-sensory level has documented effects on spatial cognition (Utz et al., 2010; Wilkinson et al., 2014), activates a wide network of multi-sensory cortical areas (Stephan et al., 2005; Stephan et al., 2009) and may introduce plastic changes that are similar to those of TDCS (Utz et al., 2010). Depending on the montage, the concomitant stimulation of other cranial nerves is also possible (e.g., vagal nerve and optic nerve).

In summary, the concomitant stimulation of cranial nerves and sensory organs can complicate the demonstration of structure–function relationships for both behavioral and physiological effects of TES: Behavioral effects, such as shifts in visual attention, can be influenced by nerve stimulation, and the physiological BOLD changes measured after DC stimulation of nerves are similar to those seen after stimulation of cortical targets (Lang et al., 2005; Stephan et al., 2005, 2009, Polania et al., 2012). Despite the similar therapeutic effects (Fitzpatrick and Day, 2004; Guleyupoglu et al., 2013; Shiozawa et al., 2014), the field of electric nerve stimulation is seldom connected to transcranial brain stimulation and we are not aware of a study directly comparing peripheral and transcranial stimulation methods. It can, however, be speculated that some of the behavioral/therapeutic effects attributed to the direct cortical effects of TES may be mediated *via* indirect input through afferent nerves and sensory organs.

## Transcranial Alternating Current Stimulation

While rarely discussed for TDCS, the problem of indirect brain stimulation is better recognized for TACS. It is known that alternating current stimulation of the retina induces the visual perception of phosphenes (Schutter, 2016). While TACS-induced phosphenes were initially thought to be solely caused by the stimulation of the primary visual cortex (Kanai, 2008), a row of studies has demonstrated that volume conduction in the head allows for direct retinal stimulation for a wide range of montages, even for those that are not targeting visual areas (Schwiedrzik, 2009; Schutter and Hortensius, 2010; Kar and Krekelberg, 2012). The direct activation of the retina through TACS is also suggested by electric field modeling demonstrating that, depending on montage, stimulation intensities as low as 500 μA may result in sufficiently strong electric fields in the retina (Laakso and Hirata, 2013).

Retinal phosphenes present a general problem for studies trying to link a brain function to the specific frequency of the TACS intervention in the presumed cortical target area. Experiments in the cat visual cortex have shown that pulsed visual stimulation, similar to TACS-induced phosphenes, can entrain neuronal assemblies in the visual cortex *via* the retino-thalamic pathway. Entrainment may not only be observable in the stimulated frequency but also in first- and second-order harmonics and throughout many areas of the visual system (Gray and McCormick, 1996). Also, human studies indicate that pulsating visual input can entrain EEG oscillations (Robinson, 1983) and that this effect is not confined to the early visual cortex. In fact, pulsed visual stimulation in the alpha, beta, and gamma frequency range has been shown to modulate excitability of the primary motor cortex (Strigaro et al., 2013) and also impacts cognitive performance: Williams and co-workers (Williams, 2001) demonstrated that words could be better remembered when they were preceded by a small flickering stimulus at 10.0 Hz. The effect was frequency specific as other frequencies (8 Hz, 11.7 Hz) did not induce this effect. This effect can even be observed for sub-consciously perceived pulsed visual stimulation and impacts cognitive performance in patients and healthy volunteers (Knez, 2014). Additionally, the effect of visual flickers scales with the distance of the flicker frequency to the individual alpha peak frequency (Gulbinaite et al., 2017), indicating that the behavioral effect of flickering light is indeed based on indirect entrainment of endogenous brain oscillations through the retino-thalamic pathway.

The fact that retinal phosphenes are perceived during TACS and not during TDCS is not due to differences in retinal current flow or field strength between the techniques but has a physiological origin. The ganglion cells of the retina form highly sensitive receptive fields as they are tuned to fire at either onset or offset of a stimulus. The accentuation of both beginning and end of a stimulus emphasizes stimuli that change over time, making the retina more susceptible to AC when compared with DC stimulation (Meister and Berry, 1999).

While indirect brain stimulations *via* afferent nerves can present a challenge for the demonstration of unambiguous structure–function relationships demonstrated with TACS and TDCS, there are several ways to minimize or control the expected contribution of indirect cortex stimulation. One option that has been shown to reduce sensory side effects of concomitant nerve stimulation is the use of more focal pseudo-monopolar montages (Heise et al., 2016), as their focality decreases the extent of extra-cortical tissue stimulation. The use of electric field modeling tools is also recommended as they allow the researcher to estimate the focality of their chosen montage. However, most existing head models do not include high-definition segmentations of extracranial tissue and are therefore not ideal in predicting the amount of extra-cortical stimulation. While focal montages may reduce sensory side effects, they do not mitigate the issue completely, and there may be stimulation locations where significant concurrent nerve stimulation is hard to avoid. Here, active control conditions are additionally needed to match the contribution of peripheral co-stimulation.

To reduce cutaneous stimulation directly under the electrode, several studies have suggested the application of a topical anesthetic (DaSilva et al., 2011; McFadden et al., 2011). This can successfully decrease the tingling and burning sensations associated with TES and can be recommended for better blinding but it should be borne in mind that topical anesthetics are not likely to decrease the amount of indirect brain stimulation *via* the non-cutaneous sensory routes discussed above.

The use of focal electrode montages informed by electrical field modeling could be combined to minimize peripheral co-stimulation. A recent study by Khatoun et al. (2018) compared the effectiveness of standard and focal TACS montages to entrain physiological tremor and elicit phosphenes. The standard bipolar montages used a peak amplitude of 1.9 mA and an extra-cephalic return electrode to target either the prefrontal cortex and M1, respectively, while the high-current focal stimulation employed a 4 × 1 montage at 4.5 mA centered over M1-HAND. They found that only the focal montage over M1 entrained physiological tremor without eliciting phosphenes. Both bipolar montages elicited phosphenes and entrained tremor, rendering it possible that the tremor may have been mediated *via* the phosphenes rather *via* a direct modulation of specific brain areas. Finally, an additional control experiment applying high-current focal stimulation to the occipital cortex failed to elicit phosphenes, suggesting that retinal stimulation was causing the phosphenes in the non-focal montages. Khatoun et al. (2018) used electric field modeling to visualize the focality of montages. The study by Khatoun et al. (2018) illustrates the inherent ambiguity when interpreting TES-related behavioral modulation using non-focal montages. The study also introduces strategies to minimize and estimate the impact of non-cortical stimulation.

## Non-Focal Cortical Current Distributions

A necessary but not sufficient prerequisite for establishing a causal relationship between brain function and structure is to ensure that TES reliably and selectively targets the cortical area of interest. For bipolar TES, the currents injected are characterized by non-focal and inhomogeneous field distributions, resulting in a speckled stimulation of several areas (Opitz et al., 2015). Building on the “virtual lesion concept” that has been successfully applied in transcranial magnetic stimulation (TMS) studies (Walsh and Cowey, 2000), investigators often base their interpretation of TES effects on the assumption that the area under the stimulation electrode receives the highest current densities. Accordingly, any behavioral or physiological change associated with stimulation of the supposed target area is taken as evidence for a causal link between the target area and the brain function under investigation.

TES is regularly referred to as less focal than TMS, but this often seems to refer primarily to the size of the electrodes. However, there are two factors that impact the focality beyond electrode size. First, the distance between anodal and cathodal electrode(s) determines how much TES spreads into the brain; the larger the distance between the two electrodes, the larger the brain volume that is targeted by TES (Faria et al., 2011). Second, TES does often not result in a homogenous stimulation of a cortical region. The notion that TDCS results in homogenous anodal and cathodal stimulation is an oversimplification; similarly, the notion that TACS entrains entire brain areas into the same or opposite current phase is also oversimplified.

The statement that the distance between “active” and “return” electrode influences the spatial extent of stimulated structures sounds trivial but is often neglected. It has been argued that the use of large return electrodes “dilutes” the currents at the “return” site and thereby focalizes stimulation under the active electrode (Nitsche et al., 2007). Other attempts to avoid a cortical effect at the return electrode commonly include the placement of the return electrode at an extra-cephalic position (Nitsche et al., 2007). However, electric field models suggest that neither of these strategies can secure that stimulation is strongest at the intended target site (Datta et al., 2009; Saturnino et al., 2015). Computational models are important for estimating TES effects, as many variables, including technical (electrode thickness, size, gel/saline) and anatomical (cortical folding, corticospinal fluid, skull thickness) factors, influence the current flow and make intuitive estimations of the current flow patterns bound to fail regularly (Opitz et al., 2015; Saturnino et al., 2015). In the same vein, brain mapping studies often failed to pinpoint TES-related effects in the brain area under the “active” stimulation electrode, but rather report effects in remote brain regions (Lang et al., 2005; Cabral-Calderin et al., 2016; Antal et al., 2017). Functional effects have also been observed under the return electrode (Heinrichs-Graham et al., 2017). These considerations question the possibility to infer a clear structure–function relationship with behavioral TES studies without employing functional brain mapping, as all brain structures between the anode and cathode may be stimulated and confer behavioral changes in experimental tasks.

The use of brain mapping studies is crucial when trying to capture how much the effects of TES spread across the brain. However, the demonstration of remote effects does not imply that TES causes non-focal brain stimulation. Remote TES effects can be caused through projections from the primary cortical target site to remote brain regions (Yu et al., 2015; Bergmann et al., 2016). A spread of stimulation effects within functional brain networks also explains why several nodes of the same network constitute therapeutic TES targets for the same disease (Fox et al., 2014). Based on functional neuroimaging alone, it is not possible to distinguish among indirect network effects evoked by spread through the targeted network, indirect brain stimulation *via* stimulated sensory organs, and direct stimulation of remote brain regions arising from non-focal currents. Knowledge about the brain’s connectome and about the electric field distributions in the brain might help to resolve this ambiguity.

A recent study by Jones et al. (2015) illustrates the inherent problem in terms of functional localization and the additional value of field simulations. In that study, bipolar TDCS was given through an extra-cephalic cathodal electrode over the contralateral cheek with a parietal (group1) or a frontal (group2) anodal electrode to boost working memory (WM). The authors showed that both montages improve WM when compared with sham while not being significantly different from each other. While it is plausible to assume that WM is similarly boosted by parietal and frontal stimulation, the authors cannot exclude that the similarities between montages stem from activations of deeper brain areas caused by the common return electrode. In fact, based on their FEM simulations, the authors state that all tested montages also cause high electric fields in the temporal pole. The temporal pole has been associated with several aspects of visual WM such as object and location matching (Ungerleider et al., 1998), emotional processing and object naming and comprehension Bonner and Price (2013). These contributions of the temporal pole to WM may have contributed to the TES-induced performance changes in the experimental task (Olson et al., 2007; Tsapkini et al., 2011).

The interpretation of the current direction is also more complex than generally assumed. Most studies presume that cortical structures close to the anodal electrode are subjected to homogeneous anodal (inward-flowing) current while structures close to the cathode receive a homogeneous cathodal (outward-flowing) current. This assumption is based on studies in species with a smooth cortical surface (Bindman et al., 1964; Purpura and McMurtry, 1965). However, the folded cortex of the human brain causes a speckled pattern of inward- and outward-flowing currents across gyri (Datta et al., 2009; Reato et al., 2013; Saturnino et al., 2015). This complicates the differentiation into brain areas receiving mainly anodal or cathodal stimulation. A recent study investigated the effect of current flow in relationship to cortical folding (Rawji et al., 2018) and showed that electrical currents perpendicular to the cortical surface (leading to current in- or out-flow) had greater effect on MEPs than currents flowing horizontally within the cortical sheet. The authors recommended including the current orientation with respect to cortical folding of the target areas as a variable for individual placement and dosage.

Can bipolar TES be used to link cognitive performance to specific neural structures? To address this question, we modeled the TES-induced electric fields for some recent papers to link cognitive performance to specific neural structures. Electric field modeling based on numerical methods and realistic head models is increasingly used by the community for planning and analysis of TES and TMS interventions and offers an invaluable tool for understanding current distribution in the brain during TES. Physical validation of electric field models and demonstration of their predictive power for the physiological stimulation effects is paramount, and in the recent years, a growing number of studies have compared the model-predicted electric fields of TMS and TES with physical measurements and physiological markers (Thielscher et al., 2011; Datta et al., 2013). They have demonstrated that electric field models have considerable predictive power of physiological effects (Opitz et al., 2013, 2014; Bungert et al., 2017; Laakso et al., 2018, Mikkonen et al., 2018) and that the simulated electric fields correlate with measured electric fields or current density in the brain (Opitz et al., 2016, 2018; Huang et al., 2017, Goksu et al., 2018). While additional validation is important, the current data suggest that electric field models indeed offer a valuable tool for understanding current distributions induced by TES.

**Figure 1:**
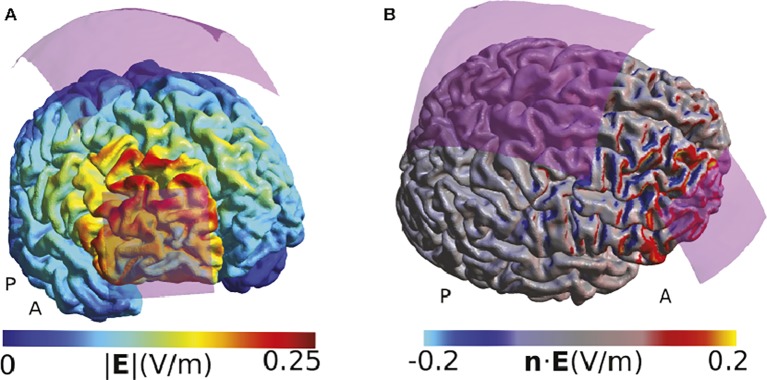
Simulation of the TDCS electric field for the montage used by Raja Beharelle et al. (2015). **(A)** Visualization of the electric field strength (i.e., the norm or length of the electric field vectors). Strong fields are located at the edge of the electrode and adjacent to the electrode, complicating exact functional localization of effects. **(B)** Display of the component of the electric field that is directed perpendicularly to the cortex surface (i.e., the normal component of the field). Positive values indicate a field flowing into the cortex, and negative values indicate a field flowing out of the cortex. The cortical folding causes a speckled pattern of the field distribution, with currents often entering a gyrus on one side and leaving it on the other side. All simulations were done using SimNIBS 2.1 and the included “Ernie” example dataset. The anode was modeled as a 5 × 5 cm electrode and the cathode as a 10 × 10 cm electrode as described by Raja Beharelle et al. (2015). The anode was placed above the rPFC, defined using the MNI coordinates given in that paper, and the cathode was placed at the Cz position of the EEG 10/20 system. The current strength was set to 1 mA. Both electrodes were assumed to consist of thin rubber layers placed over 5 mm of conductive gel. The fields are shown in the middle cortical layer, located halfway between the gray and white matter surfaces.

We have chosen the studies where we modeled the TES-induced electric fields as representative examples to illustrate how computational models can be used to aid the interpretation of behavioral or physiological TES effects. In a study from 2015, Raja Beharelle et al. (2015) used a montage with a small active electrode over the frontopolar cortex and a large electrode over the vertex to explore the role of the right frontopolar cortex on decision-making involving exploitation-exploration trade-offs during a virtual slot-machine game. They state that using a large return electrode made them “certain that the effects of TDCS on exploratory behavior would not be affected by neuromodulatory influences on neural activity under the reference electrode,” concluding that all behavioral effects observed were only caused by stimulation of the frontopolar cortex (Raja Beharelle et al., 2015). Our field simulations suggest that the effects directly under the return electrode are indeed relatively weak (Figure 1). However, the montage affects large parts of the right frontal lobe, including the premotor cortex and the inferior frontal areas. These areas all receive comparable field strength, and the maximal peak of stimulation was not under the active electrode but dorsally adjacent to it. While our modeling is restricted to a single example head model, it suggests that the applied montage cannot convincingly localize the right frontopolar cortex as the neural structure underlying the behavioral effects.

**Figure 2:**
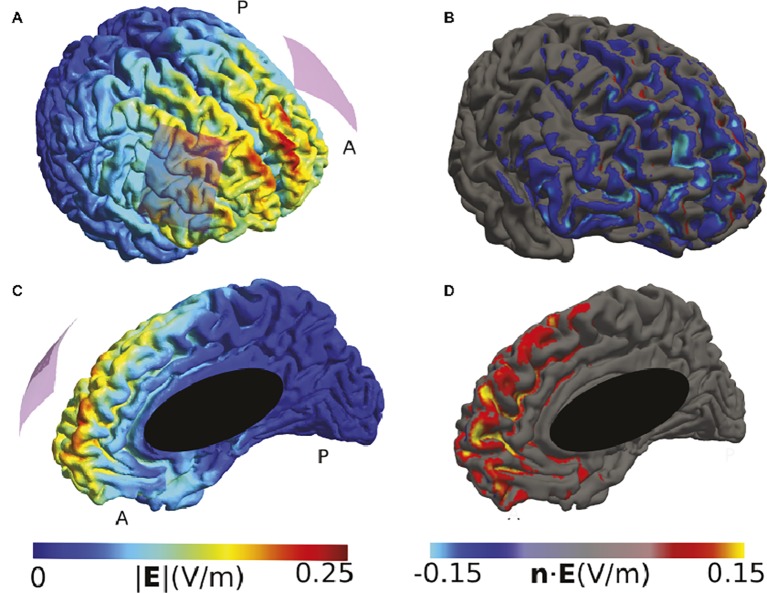
Simulation of the TDCS electric field caused by the montage described in (Conson et al., 2015). **(A)** and **(C)** High electric field strengths are located at the edges of the electrodes and in between them, including frontal midline brain areas. **(B)** and **(D)** The normal component at frontal midline structures of the right hemisphere tends to have the opposite polarity compared to large parts of the cortical surface underlying the right electrode. Both electrodes were 5 × 7 cm^2^ sponge electrodes of 8 mm thickness, with a 4 × 5 cm^2^ silicon rubber layer inside. The anode was placed above F3, and the cathode above F4 of the EEG 10/20 system. The current was set to 1 mA.

Many montages also use a bi-hemispheric montage in which anode and cathode are placed over corresponding cortical targets in each hemisphere. To illustrate such a montage, we have modeled a study that stimulated the bilateral dorsolateral prefrontal cortex (DLPFC) to show that the anodal right/cathodal left montage modulated the response to fearful faces (Conson et al., 2015). Our modeling suggests that the highest current densities were reached close to the midline in the medial frontal cortex and not under the electrodes (Figure 2). The involvement of midline areas is relatively common for bi-hemispheric montages. It indicates that, in this example, the location of fear modulation cannot be unambiguously attributed to the DLPFC.

**Figure 3:**
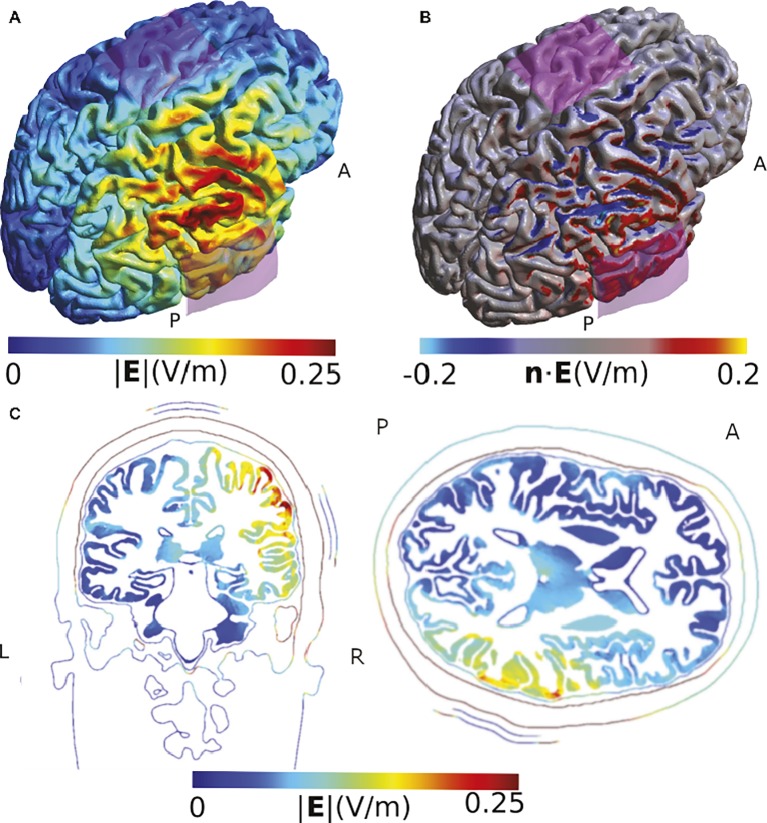
Simulation of the TDCS electric field obtained using the montage described by Sowden et al. (2015). **(A)** Electric field strength. **(B)** Normal component of the electric field. The two sides of the gyrus of the temporo-parietal junction are polarized in opposite ways. **(C)** Slice view of the electrical field strength. While this view has the advantage of visualizing field strength in deeper cortical and subcortical areas such as insula, putamen, and thalamus, it does not allow displaying current direction relative to the cortical surface and misses the general overview that visualizations on the rendered brain surface offer. Electrodes were modeled using the same shape parameters as for Figure 2. The anode was placed above CP6 and the cathode over Cz. The current was set to 1 mA.

In a final example, we illustrate the impact of the cortical folding on the direction of the stimulation effect, using a study of Sowden et al. (2015). Similar to previous simulation results, our model suggests that the highest field strength occurs in areas adjacent to, but not directly under, the electrode. It also illustrates that the gyrus of the targeted temporoparietal junction receives an inflowing (anodal) current on one side, but an outflowing (cathodal) current on the other side (Figure 3). While this does not challenge the validity of the observed behavioral effect of the specific montage on lie detection, it suggests that the authors are right in carefully refraining from making assumptions about the exact mechanism of action within the stimulated areas.

## Concurrent Stimulation of Several Brain Areas

A special case that further complicates the unambiguous demonstration of structure–function relationships is multi-site stimulation by TES. Especially, TACS has been used for targeting two areas to manipulate the temporal phase relationship between the local oscillations in those areas (Struber et al., 2014; Polania et al., 2015). However, the employed montages can result in rather complex spatial simulation patterns that differ between in- and out-of-phase stimulation in large parts of the brain. Modeling data from our group have demonstrated that only focal montages like the ring-montage or the high-definition montage allow the targeted manipulation of the phase relationship between the two target areas without injecting unwanted electrical fields in other areas (Saturnino et al., 2017).

## Dose–Response Relationships

While animal models inform our understanding about the basic mechanism-of-action of TES (Bindman et al., 1964; Purpura and McMurtry, 1965), they can hardly be used to draw inferences about dosing in human applications as they often apply much higher intensities directly to the exposed cortical surface (Jackson et al., 2016). Computational models and intracranial recordings agree that the field strength reached at the target site in human TES studies is between 0.2 and 0.5 V/m for a stimulation intensity of 1 mA, scaling up to field strength around 0.8 V/m for stimulation intensities around 2 mA (Datta et al., 2009; Opitz et al., 2016; Huang et al., 2017). Field strengths of this magnitude are thought to be at the low edge of the intensities needed for generating measurable physiological effects in neurons (Jefferys et al., 2003; Ozen et al., 2010; Reato et al., 2010; Huang et al., 2017). Electric fields in the brain are relatively weak and variable because skin, skull, and subcutaneous soft tissue act as a shunt and divert a significant part of the injected current away from the brain (Datta et al., 2009). A recent *ex vivo* study indicated that the skull and soft tissue surrounding the brain shunt around 60–75% of the injected currents away from the brain (Voroslakos et al., 2018). According to this study, the current reaching the brain in conventional TES montages is too low to temporally bias neuronal spiking, but may affect the brain through stochastic or rhythmic resonance (de Berker et al., 2013). The authors suggest that scalp currents of at least 6 mA (three to six times the currently applied dosage) would be needed to drive cortical spiking in humans. *Ex vivo* results are not directly transferable to *in vivo* TES studies due to changes in conductivity after death (Opitz et al., 2017) but also *in vivo* studies have to assume that a significant portion of the injected current is shunted through the skin, indicating that potentially higher current strengths may be required for reliable direct brain stimulation. However, simply increasing the currents injected through conventional montages comes with its own set of problems as this additionally increases the non-focality of TES, both due to increasing effects of peripheral co-stimulation and more widespread current distributions. The demonstrated nonlinear dose–response relationships documented for low current strength (0–2 mA) further indicate that simply increasing the intensity of stimulation may not necessarily improve the efficacy of TES (Batsikadze et al., 2013; Jamil et al., 2017).

Recent years have witnessed promising technical developments that have the potential to deliver stronger currents to the brain without increasing peripheral stimulation while increasing focality. These approaches apply multi-electrode montages and assume that transmembrane polarization sums up where the electric fields overlap in the brain. Temporal interference stimulation uses high-frequency electrical currents that are outside of the neurophysiological dynamic range. The currents of two or more electrode pairs are slightly shifted in frequency so that they modulate each other in a way that the emerging envelope frequency, determined by the difference of the base frequencies, is in the dynamic range of neural firing (Grossman et al., 2017). Alternatively, intersectional short pulse stimulation gives extremely short intersectional pulses though multiple electrode pairs and tries to exploit the temporal integration property of neurons. If successive electric fields overlap in a neuronal population during the window of temporal integration (approx. 30 ms), they are assumed to sum up where the fields overlap (Voroslakos et al., 2018). Both techniques have the potential to increase the electric fields in the brain relatively focally and without increasing peripheral effects. However, they have so far only been demonstrated in rodents.

In summary, we have discussed that non-specific effects of peripheral co-stimulation and the low spatial focality of TES complicate the establishment of structure–function relationships between the targeted area and the observed behavioral or physiological effect. The uncertainty in identifying the primary site of action limits the usefulness of standard bipolar TES as an investigational method in the cognitive neurosciences and may also contribute to the observed variability in reported results. Future studies seeking to stimulate cortical target areas should include electric field modeling into their experimental design to estimate target engagement and choose the electrode montage most suited for their purpose. In particular, the use of more focal pseudo-unipolar ring and 4 × 1 montages or computationally optimized multi-channel arrangements can help to focalize the stimulated area and minimize unwanted peripheral co-stimulation (Dmochowski et al., 2011; Edwards et al., 2013; Villamar et al., 2013). To be able to better control for peripheral co-stimulation, electric field modeling should include improved models of extra-cranial tissue to more accurately estimate shunting and peripheral co-stimulation. In addition, experimenters may emphasize the use of carefully designed “active” control conditions that seek to match also sub-threshold peripheral co-stimulation. Continued interest to map TES-induced changes on whole-brain levels by combining TES with neuroimaging tools such as EEG (Liang et al., 2014; Bergmann et al., 2016) and fMRI (Saiote et al., 2013; Hartwigsen et al., 2015) will help to demonstrate target engagement and to further understand the changes induced by TES on a brain-circuit level. Finally, in the future, the use of novel multi-electrode approaches may make it possible to induce stronger electric fields at focal cortical targets and less unwanted co-stimulation.

## Author Contributions

AK, AT and HS have developed the idea and written the manuscript. GS has calculated the field calculation and commented on the manuscript.

### Conflict of Interest Statement

HS has received honoraria as speaker and consultant from Sanofi Genzyme, Denmark, and as senior editor (NeuroImage) from Elsevier Publishers, Amsterdam, the Netherlands, royalties as book editor from Springer Publishers, Stuttgart, and a research fund from Biogen Idec, Denmark.

The remaining authors declare that the research was conducted in the absence of any commercial or financial relationships that could be construed as a potential conflict of interest.
